# COVID-19, Telecommuting, and (Virtual) Sickness Presenteeism: Working From Home While Ill During a Pandemic

**DOI:** 10.3389/fpsyg.2021.734106

**Published:** 2021-10-15

**Authors:** Sascha Alexander Ruhle, René Schmoll

**Affiliations:** Faculty of Business Administration and Economics, Heinrich Heine University Düsseldorf, Düsseldorf, Germany

**Keywords:** presenteeism at work, COVID-19 pandemic, telecommuting, telework, remote work, qualitative analysis

## Abstract

This study explored (virtual) sickness presenteeism in the context of the COVID-19 pandemic. Using qualitative data from 505 members of the German working population, it investigates how working from home, which rapidly increased because of the COVID-19 outbreak, is perceived with regard to the pandemic. The study explored how this development affects the decision to show absence or presence in case of illness. More than 1,300 responses to different open-end questions by presenteeists and non-presenteeists were analyzed. The findings suggest that many previously identified reasons for deciding for or against presenteeism are still applicable. However, noteworthy differences with regard to both telecommuting and the pandemic occurred. Virtual sickness presenteeism seems to be strongly encouraged by the possibility to adjust working conditions at home. Additionally, COVID-19 has affected the perceptions of health at work. The study contributes to a more in-depth understanding of (virtual) sickness presenteeism during a global pandemic. Six propositions for future research are developed, and the importance of context for the consequences of virtual sickness presenteeism is discussed.

## Introduction

The COVID-19 outbreak has led to many changes in the workplace for millions of workers. Notable among them is that the threat of contagious viral disease has impacted feelings of security and health (Ahorsu et al., 2020), thereby changing the behavior of individuals. In addition, many individuals had to move away from a central office to work from home on an unprecedented scale (Kniffin et al., 2020). Organizations and employees barely had time to prepare for these changes (De' et al., 2020). Both changes have impacted individuals in terms of their attendance behavior at work, as perceptions of health and illness changed and telecommuting was suddenly allowed even where it was previously forbidden.

This is especially important for research on presenteeism, defined as the behavior of working in the state of ill-health (Ruhle et al., 2020), as it impacts what attendance behavior can be considered acceptable. Before the pandemic, attending work while fighting a cold was often accepted. However, research has found that major changes in the workplace caused by affective events (Mignonac and Herrbach, 2004) or changes in social norms (van Kleef et al., 2019) can impact perceptions of acceptable behavior. Although knowledge regarding the formation of presenteeism is steadily increasing (Johns, 2010; Miraglia and Johns, 2016; Lohaus and Habermann, 2019), our understanding of presenteeism during a pandemic, particularly while telecommuting, is lacking.

Therefore, understanding the impact of the COVID-19 pandemic and the respective changes in the perceptions of acceptable presenteeism behavior is necessary because some of the changes the pandemic has caused will only be partially reversed, and the transition back to a potential new “normal” will be slow (Rigotti et al., 2021). For example, forecasts suggest that, after the pandemic, 25–30% of the workforce will continue to telecommute multiple days per week (Global Workplace Analytics, 2021). In addition, employees who work from home show presenteeism more frequently than employees who work on-site (Steidelmüller et al., 2020), but the mechanisms that underpin this effect remain unclear. Furthermore, little is known about how the pandemic has impacted the decision process that (telecommuting) employees undertake when they are ill. The choice between virtual sickness presenteeism, “regular” sickness presenteeism, and sickness absenteeism might depend on the experience of individuals with the crisis, where they work, and how these two factors interact.

In approaching this issue, we focus on three major research questions (RQ). First, we ask how telecommuting affects the decision to show absence or presence in case of illness (RQ1). Given that physically attending work is currently and often no longer mandatory or possible, we seek to determine how individuals decide for one behavior and the consequences that may result from such. Second, we analyze how the COVID-19 pandemic affects the perception of sickness presenteeism (RQ2). Third, we examine how these perceptions, in combination with telecommuting during the pandemic, impact individuals and organizations (RQ3). Clarifying the changes in the understanding of individuals and organizations of acceptable behavior can be useful for organizations that must deal with both workers who work from home and those who return to the workplace.

This research contributes to the field of presenteeism at work, especially regarding the perceptions of sickness presenteeism and its legitimacy or norms in combination with the role of telecommuting and the impact of the pandemic. First, we aim to gain a deeper understanding of the mechanisms that might explain why individuals choose presenteeism over absenteeism when working from home while ill (Ruhle et al., 2020; Steidelmüller et al., 2020). Second, we contribute to explaining how sickness presenteeism may be a dangerous behavior during a pandemic (Eisen, 2020). As sickness presenteeism is evident during this time (van Der Feltz-Cornelis et al., 2020), we provide, to the best of our knowledge, the first analysis of the role of the pandemic in the decision to choose sickness presenteeism over absenteeism. Third, we provide avenues for future research, especially concerning the consequences of virtual sickness presenteeism. Furthermore, we look into the link between telecommuting and health-related behaviors, a topic that is still under-researched (Allen et al., 2015).

## Conceptual Background

### Sickness Presenteeism

Miraglia and Johns (2016) merged the increasing research on sickness presenteeism into a dual-path model. They systematized and described two avenues that impact the decision to choose sickness presenteeism over sickness absenteeism, namely, individual health, which decreases sickness presenteeism, and job satisfaction, which promotes sickness presenteeism. However, the formation is more complex, as both contextual and individual antecedents must be considered (Johns, 2010). The distinction between the decision process of individuals and their respective health-related vulnerability must also be considered. While the decision process encompasses various reasons for presenteeism, health problems may affect the probability of having to choose between absence and presence (Ruhle et al., 2020) since ill-health creates decision-making situations more often among those who are relatively ill than it does among those who are relatively healthy. Consequently, reasons frequently reported for sickness presenteeism are multilayered and interrelated. It is beyond the objectives of this study to describe the various causes of presenteeism in general, which can be found elsewhere (Johns, 2010; Lu et al., 2013; Knani et al., 2018; Lohaus and Habermann, 2019; Ruhle et al., 2020). Instead, we focus on *job demands*, such as heavy workload, understaffing, or overtime, *working arrangements*, such as shift work or excessive working hours along with *job resources*, such as job design, job control, and interpersonal factors that impact sickness presenteeism and are relevant for virtual sickness presenteeism (Ruhle et al., 2020). These aspects are highly relevant for the decision to show sickness presenteeism in the workplace (Miraglia and Johns, 2016). Furthermore, the extant research on sickness presenteeism has asked for qualitative research that can help to clarify the path dynamics in choosing presenteeism vs. absenteeism, especially the tradeoffs that individuals consider when they make this decision (Miraglia and Johns, 2016), for which the selected aspects provide a fruitful starting point.

In addition, the nature of the consequences of sickness presenteeism has recently been challenged. While sickness presenteeism is negatively related to self-reported productivity loss (Schultz and Edington, 2007; Zhang et al., 2010) and a downturn in health (Skagen and Collins, 2016), Karanika-Murray and Biron (2020) proposed the need for a more fine-grained understanding of the consequences of sickness presenteeism. For example, based on the conservation of resources theory, they proposed that functional presenteeism could allow for an ideal adjustment of health-related constraints in relation to performance demands, which would not automatically result in productivity loss or health impairment. They proposed that sickness presenteeism is a process of adaptation in which individuals draw on available resources, such as job control and adjustment latitude, to balance health and performance (Karanika-Murray and Biron, 2020).

### Telecommuting

One way for individuals to enrich their resource pool is telecommuting (also referred to as telework or remote work). It is a work arrangement that allows at least a portion of the job of a worker to be accomplished away from a central workplace, typically from home, using technology to interact with others (Allen et al., 2015). Thus, there is no need to commute to the central workplace to work. However, telecommuting is more complex, as arguments for telecommuting as both a job resource and a job demand should be considered.

On the one hand, telecommuting may function as a job resource. As a major benefit, the ability of telecommuters to adjust their working activities and time to meet their own needs and desires may increase (Golden and Veiga, 2005; Gajendran and Harrison, 2007). In particular, the literature is spurred by the notion that telecommuting may provide individuals with the flexibility they need to address the demands of both work and family (Allen et al., 2015). Meta-analytic evidence has supported this notion and suggested the beneficial effects of telecommuting on work-related outcomes like organizational commitment, job satisfaction, job performance, turnover intention, and role stress (Gajendran and Harrison, 2007; Harker Martin and MacDonnell, 2012; Allen et al., 2013).

On the other hand, telecommuting may also function as a job demand. The same flexibility that allows telecommuters to adjust their work enables them to extend their work into non-work time and non-work spaces, which are normally reserved for private life (Schlachter et al., 2018; Schmoll, 2019). In addition, extensive telecommuting has the potential to increase professional isolation and reduce the relational life of an individual (Buomprisco et al., 2021). Telecommuting also enables work to be relocated from an office desk to non-ergonomic workstations like a couch or a bed (Davis et al., 2020). These issues may have detrimental implications for the health of teleworkers in the long run (Buomprisco et al., 2021).

### Sickness Presenteeism, Telecommuting, and the COVID-19 Pandemic

Against the backdrop of these developments, the COVID-19 outbreak forced a rapid change in both the perceptions of illness and the availability and, in some cases, enforcement of telecommuting (Kniffin et al., 2020). While there was very little evidence on the relationship between sickness presenteeism and telecommuting before the pandemic, research on the decision of individuals between absence and presence during a pandemic is non-existent. The few studies that have linked sickness presenteeism and telecommuting have shown that the probability of showing sickness presenteeism increases with the intensity of telecommuting (Steidelmüller et al., 2020). The authors speculate that this might be related to the circumstances in which the individual no longer has to commute to work, has no way to infect others at work, or has increased adjustment latitude. Furthermore, virtual sickness presenteeism can be considered self-endangering (Steidelmüller et al., 2020).

However, how telecommuting impacts the decision to work from home despite illness, that is, virtual presenteeism, remains unclear. Some motives for sickness presenteeism might differ significantly or might no longer be applicable when an individual telecommutes. For example, being perceived as hard-working (Simpson, 1998) might be more difficult when one is not present in the workplace, while the potential for adjustment might be greater, as the time and place of work might be partially in the hands of the employee (Steidelmüller et al., 2020). Other aspects of work, such as social support (Chen et al., 2021) or attendance pressure (Aronsson and Gustafsson, 2005) might change only slightly and be equally important.

## Materials and Methods

### Data Collection

We asked workers in the German working population about working while ill as part of a larger online survey named “working conditions during a pandemic.” Data collection took place in September and October 2020. During this period, there were no legal restrictions regarding the place of work. Participation for the study was solicited through an email message that was sent to the diverse contacts of the authors, including employees of a large university and employees of a trade union confederation. The study was approved by the data protection officer of the faculty. Conformity with the General Data Protection Regulation (GDPR) has been confirmed. A data protection declaration was presented to everyone before participation. Permission to process data was obtained from all participants. Of course, participation in the study was voluntary.

In sum, 625 individuals participated in the study, and we differentiated between 303 presenteeists, namely, those who had worked at least once while ill in the last 3 months, either virtually or on-site, and 322 non-presenteeists, particularly those who had been absent when sick or had not been sick at all. The respondents were not forced to answer every question. As a result, not all participants answered all questions, which resulted in missing data. Overall, 505 individuals commented on at least one of our questions, with 33.9% of the individuals being male, 65.1% being female, and 1% indicating “other” as their gender. One hundred ninety-nine of them were presenteeists and 306 were non-presenteeists. They spent an average of 13 working hours telecommuting and 23 h on-site and reported an average of 4.4 health events in the last 3 months. From these participants, we received 1,377 text segments with more than 30,498 words (in German).

Presenteeists answered the following open-end questions regarding their decision to choose presenteeism (P1): “Please describe why you decided to work while sick. What were the reasons for this? How did this behavior differ from your behavior before the pandemic?” We also asked about the context (P2): “Where were you working at home, the office, or somewhere else? What were the hours of work? Were there any special circumstances?” Finally, we asked about the role of other individuals and the organization in the decision of the participants (P3): “Please describe the extent to which COVID-19 has led your organization to address employee attendance and absences in the event of illness. Briefly state whether you have been treated differently in the context of working with an illness then you were before the crisis? If so, how?”

The open-end questions of the non-presenteeists addressed their perceptions of illness in the organization (N1): “Please describe the extent to which COVID-19 has impacted perceptions of illness in your organization. Do you or others behave differently than before the pandemic? How do you and your colleagues deal with illness?” We also asked them about changes in rules related to absence and presence that were due to the pandemic (N2): “To what extent were changes in the rules about attendance and absence communicated by your employer during COVID-19? What was communicated as the ‘correct’ behavior in case of illness? Were there any changes in this regard compared to before the pandemic?” Finally, we asked what a (fictional) decision regarding attendance would look like (N3): “Imagine feeling sick on a morning when you are supposed to be working from home. How do you deal with that? How would this choice differ from days when you would have to go into the office?”

### Data Analysis

Our analytical approach consisted of a deductive approach (P1 and P2) and an inductive approach (P3, N1–N3). Following the recent recommendations from Aguinis and Solarino (2019) and Pratt et al. (2020), we used structural coding for the first coding cycle. Because our questions provided a structure for the categories, we grounded our deductive analysis on that structure. We used pattern coding for the second coding cycle, which allowed us to pull a considerable amount of qualitative data together to more meaningful units (Saldaña, 2015). We coded seven main and 28 specific categories that we defined *ex-ante* based on conceptual (Lohaus and Habermann, 2019) and meta-analytic results on presenteeism (Miraglia and Johns, 2016). Furthermore, following Creswell et al. (2007), we used this information to offer propositions based on the three research questions.

First, we categorized 10% of the material, reaching intercoder reliability of 81–95% for the deductive categories. Due to this, we adjusted them only slightly (e.g., adding an “other” category). More specifically, for the deductive part of the analysis, we analyzed the reasons for showing presenteeism using the main categories, namely, *constraints on absenteeism, job demands, job resources, health status, collegial support, attitudes, reason related to telecommuting*, and *other*. To grasp the context of presenteeism, we coded the workplace as *telecommuting, on-site, both, mobile*, or *other* and the working time as *normal, flexible, more than usual*, or *less than usual*. Table 1 shows the themes, specific categories, and coding rules. For the deductive part of the analysis, the authors coded independently.

**Table 1:** Themes, specific categories, and coding rules for deductive coding.

**Main themes**	**Specific categories**	**Coding rule**
		**Individual reports…**
Constraints on absenteeism	Absence policies	absence policies as reason for presenteeism.
	Job insecurity	job insecurity as reason for presenteeism.
	Income	fear of losing income as reason for presenteeism.
	Ease of replacement	work has to be made up upon return to work.
Job demands	Role demands	workload, understaffing, supervisory duties as reason for presenteeism.
	Time demands	overtime, work hours, time pressure, or shift work as reason for presenteeism.
	Work-to-family conflict	work-to-family conflict as reason for presenteeism.
	Family-to-work conflict	family-to-work conflict as reason for presenteeism.
Job resources	adjustment latitude	the ability to adjust the work to the health impairment as reason for presenteeism.
	Decision authority	the need to make an important decision as reason for presenteeism.
	Work significance	the importance of one's job as reason for presenteeism.
Collegial support	colleagues support	that colleagues offer support in case of illness at work.
	Relationship with colleagues	that he/she would not like to endanger the relationship with colleagues by absence.
	Supervisory support	that supervisors offer support in case of illness at work.
	Relationship with supervisor	that he/she would not like to endanger the relationship with the supervisor by absence.
	Organizational support	that the organization (unspecific) offers support in case of illness at work.
	Relationship with organization	that he/she would not like to endanger the relationship with the organization by absence.
Attitudes	Job satisfaction	satisfaction with the job (overall).
	Affective commitment	commitment toward an object (individual, team, supervisor, job, customer, etc.).
	Work engagement	their own work role.
	Organizational justice	to avoid unfairness.
Workplace	Telecommuting	working from home.
	On-site	working on-site.
	Mobile	mobile working.
	Other	other working arrangement.
Working time	“Normal”	regular working hours.
	Flexible	flexible working hours.
	More	more working hours than usual.
	Less	less working hours than usual.

*Not all initially created categories were found in the data*.

For the inductive part of the analysis, we first conducted structural coding using the categories *dealing with presenteeism changed* (Yes/No), *decision differs between telecommuting and on-site* (Yes/No), *rules and regulations have changed* (Yes/No), and *presenteeism behavior differed from pre-pandemic* (Yes/No). The second part of coding was inductive and focused on the research questions. As we had no theoretical grounding, we used an open coding procedure to retain as much information as possible by discussing the answers of participants and extrapolating the respective categories. For this step, the authors coded simultaneously and collaboratively. Table 2 shows the structural codes, specific categories, and coding rules used for the first cycle of inductive coding.

**Table 2:** Structural codes, specific categories, and coding rules for the first cycle inductive coding.

**Structural codes**	**Specific code**	**Coding rule**
		**individual reports…**
Dealing with presenteeism changed	Yes	dealing has changed.
	No	dealing has not changed.
Decision differs between telecommuting and on-site	Yes No	different decision. same decision.
Rules and regulations have changed	Yes No	rules and regulations have changed. rules and regulations have not changed.
Presenteeism behavior differed from pre-pandemic	Yes No	different behavior. same behavior.

*Structural codes were inductively analyzed in a second coding cycle*.

We conducted the analysis using MAXQDA10 (Kuckartz and Rädiker, 2019). Following the discussion of Levitt et al. (2017) regarding the transparency and comprehensibility of qualitative research, we calculated Cohen's kappa when appropriate (i.e., for deductive categories) (Banerjee et al., 1999) or provided clear reasoning for using simultaneous collaborative coding and discussed the results (Kuckartz and Rädiker, 2019). The intercoder reliability of the coding of the deductive categories averaged 88% for the codes of working time and 80% for context. For the first cycle of structural coding, Cohen's kappa ranged between 0.86 for *decision differs between telecommuting and on-site* and 0.61 *for changes in rules and regulations*, which can be considered satisfactory to perfect agreement (Burla et al., 2008). For the sake of transparency, we translated the extracts displayed from German into English and used the abbreviation P### for presenteeists and N### for non-presenteeists, which are displayed in brackets after any quotation.

## Findings

### Sickness Presenteeism in the Context of Telecommuting

*Workplace*. Our analysis of the contextual conditions of presenteeism showed that 171 individuals who reported sickness presenteeism provided us with information on the workplace, 117 (68.4%) stated that the sickness presenteeism happened while telecommuting, while 47 (27.5%) reported that it happened on-site. Only four (2.3%) reported that it happened while partially telecommuting and partially on-site and three (1.8%) were neither at home nor on-site (e.g., external meeting with a customer). It should be noted that, during the time of the data collection, there were no legal restrictions that forced individuals to work from home.

*Working time*. One hundred twenty-two of the 303 presenteeists provided information regarding their working time for the last day they showed presenteeism. Sixty-six (54.09%) reported a “normal” working time, while 22 (18.03%) reported a flexible work time [e.g., “*Flexible working hours (in total* ~*6 h, spread over* ~*10 h), with plenty of time to rest in between*” (P689)]. With regard to the volume of work, 34 individuals (27.87%) reported that they worked *less than usual*, and only one individual (0.82%) reported *more work than usual* [e.g., “*Because of weekly deadlines, I even worked on Sunday evening […]*” (P584)].

### Reasons Reported for Sickness Presenteeism

*Constraints on absenteeism*. The most prevalent constraint of absenteeism that the presenteeists reported was the ease of replacement. For our coding, this was defined as the awareness that individuals are not easily replaced at their jobs and that work piles up until their return (Aronsson et al., 2000). This antecedent of presenteeism was unaffected by virtuality, which was to be expected, as the fundamental nature of work was unaffected for most workers even though the circumstances around their work had changed. As one participant described it, “*I oversee my area completely on my own, so work that I don't do just piles up higher and higher and there's no one to do it but me*” (P517). Only scattered remarks on other restrictions were made, which is interesting with regard to the pandemic. For example, we expected an increase in the importance of job insecurity because of the insecurity that the pandemic has created (Wilson et al., 2020), but that was not the case for the vast majority of our participants. While only four participants mentioned rules and regulations, the policies related to absence were important predictors of the attendance decision in these cases, tipping the decision in favor of presenteeism, even as avoiding presenteeism and the importance of health was omnipresent during the pandemic: “*In our company, there is a time deduction for illness [absence], which makes you think twice [about calling in sick]*” (P413).

*Job demands*. Individuals reported a wide range of demands related to their roles that justified presenteeism. In line with previous results, these role demands were rooted in the careers of the participants [“*There is a lot of career pressure and you can't just stop working”* (P525)], in their role in the organization [“*As a manager, I have a high level of responsibility for a relatively large team and feel that I have a duty to continue working in the event of minor health restrictions (like a cold)*” (P218)], or in caring for other groups [“*My patients would have been less well-cared for otherwise*” (P915)]. Interestingly, these role demands were not further justified by the workplace or the pandemic situation. Participants seemed to perceive these demands as valid reasons for presenteeism, even during a pandemic.

*Job resources*. As proposed by previous sickness presenteeism research, job resources were also identified in the answers of presenteeists about the reasons for presenteeism. The reason named most frequently was related to adjustment latitude. This adjustment was predominantly described with regard to the opportunity to take breaks when needed [“*I can always do (my work) at my own pace and freely arrange my break times*” (P835)]. However, other kinds of adjustment were also described, such as not being forced to work at a desk, using tools to decrease feelings of being unwell, or being able to stop working quickly when feelings of being unwell reached a certain level. As one participant mentioned, “*The advantage was that I could always rest in between and have enough tea, and I didn't have to meet anyone in person and risk infecting them, and I could dress comfortably*” (P189). It is important to note that none of the on-site individuals reported having such an adjustment latitude, highlighting that telecommuting is related to a different way of adjusting, which will be further evaluated below. On-site presenteeists reported aspects of the significance of work as a reason, such as the personal importance of the work [“*It was for an exciting project in whose progress I had a great interest*” (P51)].

*Health status*. Another widespread cause reported for presenteeism was health events. Aspects of a health condition like the level of impairment [“*I felt only moderately affected*” (P178)], the perception that the health condition contained no risk of infecting others [“*Not a contagious disease*” (P130)], or that the combination of the health status and telecommuting allowed the person to work [“*Because I was telecommuting, I was able to work despite my broken foot*” (P952)] were described. Thus, the nature of the health status is an important aspect in the decision between absence and presence (Johns, 2010). However, the quality of this calculation might depend on the health literacy of the individual (Berkman et al., 2010), as we also found examples where considering a health condition without consulting a physician could be problematic, especially during a pandemic. For example, one individual reported choosing sickness presenteeism, as “*the illness was not serious (cold and headache)*,” but there was almost no way to separate these symptoms from common COVID-19 symptoms like cough, fever, and shortness of breath (Paules et al., 2020).

*Collegial support*. Three main aspects of support were reported as reasons for sickness presenteeism. First, some individuals did not want to strain their relationships with colleagues by being absent and losing support. These arguments were presented both in a positive notion, particularly as a form of wanting to support the team [“*I'm working on a very important project, and I have a great team that I still wanted to support*” (P404)], and with some using a more negative tone [“*There is a low tolerance on the team for absences or delays related to illness*” (P584)]. Second, the same strain should not be put on the relationship with the supervisor. Here, we only found negative examples [“*(I worked because) the mood between my supervisor and me was already very bad*” (P581)]. Third, some individuals reported these reasons on a more general level, referring to their relationships with the organizations [“*I consider it a privilege not to have to worry about my job and to be able to telecommute*” (P952)] or the goodwill [“*as a young employee who still wants to find her way professionally in the company, I would not like to accumulate sick days to avoid attracting negative attention”* (P100)] of the organization. However, we found only positive remarks when individuals reported that work itself was a reason for showing sickness presenteeism, as in such cases it was a “*distraction from being sick*” (P940) or “*that [working] is the second-best way for me to get out of my own head*” (P326).

*Attitudes*. Finally, we found only scattered evidence for work engagement as an attitudinal reason for choosing sickness presenteeism and no evidence for affective commitment, justice, or job satisfaction as reasons. While this result may be surprising, especially as job satisfaction is one major mediator in the dual-path model (Miraglia and Johns, 2016), this lack of evidence might be connected to the nature of satisfaction as a latent construct that is important only in the back of people's minds. As a global concept that includes various facets like salary, promotion, colleagues, supervisors, and the work itself (Judge et al., 2020), job satisfaction might not be a salient reason for sickness presenteeism.

### Reason for Sickness Presenteeism Related to Telecommuting

As most of the participants reported working from home while in poor health, some of the reasons for choosing sickness presenteeism were directly linked to telecommuting. However, both positive and negative aspects of telecommuting were mentioned and inductively coded.

In addition to the aspects mentioned above, *positively perceived aspects* of telecommuting could be identified. As there is no need to commute to the central workplace, it was a little surprising that this context-specific reason was mentioned frequently. Telecommuting removes the need to commute to work when one does not feel well. This benefit of working from home was reported to be a major reason for showing sickness presenteeism, as it allowed the individuals to remove a burden from their workday. As one participant put it, “*The reason was that I felt too sick to go out, commute, and work in the office, but not so sick that I couldn't work at home*” (P814).

Presenteeists also mentioned reasons related to an increased adjustment latitude while telecommuting frequently. For some telecommuters, increased temporal flexibility was an important reason for showing virtual sickness presenteeism, as “*while telecommuting, you can schedule your own time and take short breaks if you get tired, which would not be possible on-site*” (P543). Likewise, a health-related adjustment was reported, such as the possibility “*to withdraw if you don't feel well*” (P451) or to “*do the most important work from bed with a hot-water bottle and pills, which would not have been possible without telecommuting*” (P573).

Rather *negatively perceived aspects* of telecommuting were related to implicit expectations and increased opportunities to show sickness presenteeism. While implicit expectations refer to the perceived pressure to work from home while one is ill, as one works at home anyway and should be able to handle at least some work [“*I had the feeling that there was a subliminal expectation to take sick leave while telecommuting only in very serious cases since you are at home anyway*” (P584)], another aspect encompasses the self-endangering behavior that has been assumed to be relevant by previous research (Steidelmüller et al., 2020). For example, “*Calling in sick while telecommuting is a bigger barrier […] because I can arrange the work schedule more freely. So if I normally woke up with a migraine, I would have called in sick. Now I work the time off in the evening instead*” (P491).

### Differences in Sickness Presenteeism Behavior Based on the Pandemic Context

To determine whether the sickness presenteeism behavior during the pandemic differed from the pre-pandemic decision, we asked our participants whether they would have chosen differently before the pandemic. We found individuals who reported having made a different decision before the pandemic and individuals who reported that their decision was unaffected by the pandemic.

While many of the presenteeists did not explain why their decision to show sickness presenteeism was *unaffected*, we were able to identify some patterns. Participants reported that, despite the pandemic, their situation was unchanged, and sickness presenteeism was an acceptable behavior that had been shown previously: “*I've always worked […] when I felt slightly limited in health, so nothing has changed here compared to before the crisis*.” (P730), up to the point that showing sickness presenteeism was described as normal [“*This behavior is normal for me*” (P1000)]. Others reported that there was no difference in their behavior, as telecommuting was already their preferred way of dealing with sickness presenteeism, and the pandemic did not negatively impact this decision: “*Before the Corona crisis, I made the same [decision] if I didn't feel well: I could still work*” (P248).

The participants who reported that their decision was affected by the pandemic centered their reasoning around two major arguments. Despite choosing sickness presenteeism, most individuals reported that their decisions differed in that they decided to choose virtual sickness presenteeism instead of going to work, which would also have been their decision before the pandemic: “*Normally, if I had a common cold, I would go to the office and work normally, but because of the corona pandemic, I chose to work from home*” (P743). Those participants described the COVID-19 pandemic as a major barrier that prohibited working on-site but not working in general. When a participant had a health impairment that was not related to the pandemic, the pandemic presented additional opportunities for presenteeism. For example, one individual reported taking part “*in important video conferences despite torn ligaments. I probably wouldn't have done that otherwise, since I didn't have the option to telecommute before Corona*” (P461). As telecommuting was accepted in circumstances that had not been common in many organizations, taking part in meetings that otherwise would have been impossible in person was now possible. Overall, the COVID-19 pandemic increased opportunities for showing virtual sickness presenteeism.

### The Decision Process for Virtual Sickness Presenteeism

The following results are based on the data we received from non-presenteeists. The hypothetical question concerning the decision process helped us include the perspective of individuals who had not chosen sickness presenteeism and identify the underlying mechanisms of the decision to choose virtual sickness presenteeism.

#### Relationship Between Sickness Presenteeism and Telecommuting

We found that the participants differed concerning their understanding of whether sickness presenteeism and telecommuting are related. Some had the view that presenteeism while telecommuting is a viable *alternative to keep working* as “*in the past, you were simply sick and stayed at home. Those who are not well while telecommuting are now less likely to call in sick*” (N993). These participants reported that, if they would be sick while telecommuting, they would show *hidden sickness absenteeism*: “*I would go back to bed or rest. If I had to go to the office, I would call in sick, but I would rather not call in sick while telecommuting. Presenteeism at home is an alternative to sick leave*” (N441). Others had the view that there would be no difference between telecommuting and working on-site concerning sickness presenteeism: “*Despite working from home, I would call in sick […]. If the condition worsened, I would go to the doctor. Conclusion: I would not behave differently*” (N442).

In line with the idea that presenteeism is an adaptive behavior, individuals who perceived sickness presenteeism while telecommuting as an acceptable behavior reported that they would adjust their productivity not based on their health status but based on the expected productivity, and that was the factor that shaped their decision: “*If I can still perform, I would work from home. Only if I can no longer perform would I call in sick*” (N385). In contrast, some reported that their health is the most important predictor of their behavior and that sickness presenteeism would be an intermediate solution: “*I would telecommute and wait and see how my illness developed. If it got worse, I would go to the doctor*” (N128). This heterogeneity highlights the importance of both a person-centered approach to sickness presenteeism and the health literacy of individuals in reducing the risk of unhealthful and dysfunctional behavior.

#### Reasons for Virtual Sickness Presenteeism

The decisions of participants differed based on three major arguments related to adjustment latitude, commuting, and health status. First, participants stated that an increased adjustment latitude would allow them to opt for sickness presenteeism instead of calling in sick. *Temporal flexibility* would give them the ability to “*arrange […] working hours completely freely*” (N115) and “*take work breaks more frequently*” (N73) to suit their individual needs when they were ill.

In addition to the ability to adjust work schedules and take breaks, telecommuting also allowed participants to *adjust work volume*. Participants reported that they would be able to adjust the amount they work. If they would decide to work from home while ill, they “*would then work fewer hours*” (N772). Whether the participants would compensate for this reduced volume later or intensify their work to be more productive in less time remains unclear.

The third argument, *health-related adjustment*, reflected the perception of individuals that working from home has the benefit of taking care of health problems as they occur: “*When I feel unwell, I can take care of my health condition in parallel [to work]*” (N209). These participants would make their decision based on the possibility that they could handle light tasks and adjust their work according to their symptoms. Related to that reason was the *adjustment of the work environment* according to the needs of the individuals. As one participant described it, “*I would do some easy tasks and adapt my situation to the symptoms by, for example, using a heat cushion and lots of chamomile tea for stomach problems, taking a rest in the bathtub, or working on the couch with a blanket*” (N492). Thus, anything the individual perceives as a burden while ill, such as having no opportunity to rest, having to dress accordingly, meeting other people, and so forth, does not apply to telecommuting. Taken together, the increased adjustment latitude that telecommuting offers also led some non-presenteeists to conclude that the “*telecommuting environment is better for enduring illness than the office environment*” (N155).

While the role of health in sickness presenteeism is evident, as a health event is part of the definition of presenteeism, its role in the decision process is less clear. Many participants explained in detail that, “*depending on how sick I felt, I would likely still work while telecommuting, even if I would have called in sick on-site with the same symptoms*” (N104). The level of illness was also an important aspect of the decision as, if an illness of a participant was contagious, he or she would opt for sickness presenteeism from home: “*As long as the symptoms do not affect me too much (e.g., fever), I would probably work from home, whereas I would be more likely to call in sick at the office*” (N391). Of course, what constitutes “too much” is highly subjective.

### Sickness Presenteeism During the COVID-19 Outbreak

#### Changes in Dealing With Presenteeism

As expected, how people and organizations dealt with sickness presenteeism concerns during the pandemic differed. On the one hand, participants reported no change in behavior, which we coded as *unchanged dealing with sickness*. On the other hand, participants reported that the consideration of sickness presenteeism had changed, which led to categories related to the *nature of changes*.

Concerning the unchanged dealing with sickness presenteeism, it is surprising that some non-presenteeists reported having perceived no general changes related to sickness. They reported that neither their organizations nor the workers changed how they dealt with sickness presenteeism: “*No confrontation with that at all. No, I was not treated differently*” (N818). Others included the COVID-19 pandemic in their responses, which nonetheless did not suggest changes: “*COVID-19 has not led to any new insights from my organization [regarding dealing with presenteeism]*” (N896). Others reported only a few changes unrelated to presenteeism, often regarding minor modifications like disinfecting hands and contact surfaces: “*No change in behavior, except in compliance with hygiene rules (distance, no handshaking)*” (N794). Finally, in the few cases that reported no changes, dealing with sickness presenteeism was already in the measures that had been taken before the pandemic: “*Before COVID-19, my supervisor already mandated that individuals who felt ill should stay home and, if necessary, continue to work while telecommuting if they felt well-enough*” (N858).

Those who reported changes in dealing with sickness presenteeism reported changes that we attributed to various mechanisms. We found that some people reported an increased sensitivity to illness in light of COVID-19. While it is not surprising that the pandemic had an impact on how employees perceived illness at work, we found that individuals, teams, and whole organizations became more aware of the symptoms related to COVID-19: “*employees as a whole are classified as sick more quickly, and all symptoms that could be related to COVID-19 are taken more seriously*” (N591). As such, especially when on-site, sickness presenteeism was often behavior that was no longer acceptable. In some cases, the awareness of health in the workplace even encompassed areas that are not directly related to COVID-19, which we considered evidence of a higher sensitivity to health (impairment) in general: “*The topic of illness and disease has gained in importance. There is more talk about preventive health care and more is being done about it*” (P796).

Many participants reported measures related to COVID-19, which ranged from small measures like enforcing hand-washing and mask-wearing up to massive interventions like changes in who was allowed to be present on-site: “*Many of the workgroups now work in shifts, and masks are worn throughout the building. Not too many people are in a room at the same time, so the building generally appears to be emptier, and care is taken to maintain spacing*” (N272). These aspects of health protection were often directly linked to reducing the risks of infection and preventing the further spread of the pandemic. In some cases, even the absence and presence norms were reflected and communicated more clearly than previously as “*illness was more strongly considered a legitimate reason for not showing up*” (N574). These kinds of changes established new standards regarding attendance, which were also reflected in changes in rules and regulations.

#### Rules and Regulations

As we sought to determine how organizations dealt with the COVID-19 outbreak, we also analyzed changes in the communication of rules and regulations with regard to attendance behavior. For *changes in rules and regulations*, 130 participants reported that no changes were communicated. A deeper analysis of these statements revealed three major themes. For one group, the attendance regulations were strict from the beginning, such that, even before the COVID-19 outbreak, attending work while ill was unwanted and strictly prohibited or frowned upon, so any change in the rules and regulations was unnecessary. As one participant reported, “*The correct behavior has remained the same. Everything has been communicated, and nothing has changed*” (N448). Another group reported that, while the rules did not change, the pandemic influenced how employees dealt with the rules: “*As in most companies, there was a directive to stay home if there were even the slightest symptoms. I think that this was already in place beforehand, but it was not taken seriously by me or by most people. That changed during the crisis*” (P773). This insight has value, as prior research has shown that dealing with rules and regulations with regard to absence and presence is related to the perceived legitimacy of these rules (Johns, 2010). Therefore, the thread of the pandemic seems to have changed the perceived importance of following organizational rules concerning calling in sick. The third group did not receive any information regarding the correct behavior if they were ill and had no existing rules and regulations to draw on: “*No rules and regulations were given regarding the correct type of [attendance] behavior. Employees have to judge on their own if it would make sense to stay home*” (N457). Some participants complained that the organization was clueless and did not deal with the phenomena well, which resulted in a lack of clarity for employees: “*The organization has no strategy. There is just no information policy*” (P249). When we collected this information from the participants, the pandemic could no longer be considered a new and unforeseen threat. Thus, such missing communication was problematic for the health and safety of employees.

When a *change in rules and regulations* was reported, the participants reported that, in contrast to the pre-pandemic situation, presenteeism was no longer accepted either in general, without a reference to symptoms related to COVID-19 [“*It was clearly pointed out that you should stay at home if you feel unwell*” (N718)], or in those who have symptoms specifically related to the symptoms of COVID-19, sometimes with explicit referral to organizational actors that would help [“*In case of symptoms related to COVID-19, individuals are asked to stay at home and wait for the symptoms to disappear. If the symptoms persist and/or fever and suspected COVID-19 infection occur, consult a company physician (Betriebsarzt) beforehand and, if necessary, take a SARS-CoV-2 test*” (N847)]. Urging employees to stay at home when they have any signs of illness was the most common change because of the pandemic nature of COVID-19 and the fear of contagion.

Some organizations have even gone further and changed rules and regulations regarding office occupancy in general, such as: “*Our company increased the time window for presence in the office. The offices are to be staffed with only one person because of COVID-19. The other colleagues then work from home. According to the employer, anyone who feels ill should stay at home and see a doctor after 3 days at the latest*” (P646).

## Discussion

The results gave us evidence to answer our research questions. In the following section, we discuss our results and develop six propositions. Further research on these propositions is needed to clarify the nature and consequences of virtual sickness presenteeism.

### RQ1: How Does Telecommuting (e.g., Virtual Work) Affect the Decision to Show Sickness Presenteeism?

We found that the reasons reported for virtual sickness presenteeism were in line with previous results for on-site sickness presenteeism (Johns, 2011; Miraglia and Johns, 2016). Constraints on absenteeism, job demands, job resources, collegial support, and health status were all described for both virtual sickness presenteeism and regular sickness presenteeism. Many aspects of presenteeism are not based on the setting, as role demands might not differ much based on whether the work is done virtually or in person. Also, the perception that working in a state of ill health is perceived as beneficial for a career (Johns, 2010) might not change based on the context of sickness presenteeism. While a systematic comparison of these reasons from the theoretical and empirical perspectives is necessary to determine the relative importance of specific reasons, general transferability can be assumed.

*Proposition 1*: Many reasons that have been identified as influencing the decision to choose on-site sickness presenteeism can be transferred to virtual sickness presenteeism.

In addition to these known antecedents, we found differences between the decision to choose on-site sickness presenteeism and the decision to choose virtual sickness presenteeism. Whereas on-site workers can decide only whether to go to work or call in sick, the decision-making possibilities for individuals who can choose whether to work from home or on-site are expanded, as they can decide whether to call in sick, work from home while ill, or work on-site while ill. Sometimes, the decision is not between calling in sick or on-site sickness presenteeism, but between virtual and on-site sickness presenteeism. If the decision is between absenteeism and being present on-site (e.g., you have to work on-site and telecommuting is not allowed), many would choose sickness absenteeism. However, if it is possible to work from home, they would choose virtual sickness presenteeism.

For this decision, both presenteeists and non-presenteeists reported that several context-specific aspects of adjustment latitude are particularly relevant to their choice of virtual sickness presenteeism. Telecommuting provides more opportunities to adjust the environment according to individual health demands. More specifically, some telecommuters can alter their typical temporal work patterns, such as adjusting the timing and volume of work. We found indications that telecommuting makes those with temporal flexibility more likely to adjust their schedules and work breaks to suit their health condition. These findings reflect thoughts from the telecommuting literature that working remotely increases the ability of employees to adjust their work to meet personal needs (Golden and Veiga, 2005; Gajendran and Harrison, 2007) and contribute to explaining why individuals choose virtual sickness presenteeism over absenteeism (Ruhle et al., 2020; Steidelmüller et al., 2020). This finding also contributes to the telecommuting research that calls for considering temporal flexibility as an important contextual factor (e.g., Allen et al., 2015). Second, working from home reduces the burden of going to work on-site, which includes the effort of preparing to go to work and commuting that might be particularly burdensome when one is ill. The omission of commuting seems to be particularly relevant to the choice of virtual sickness presenteeism, as many people have to commute large distances to work on-site (Calderwood and Mitropoulos, 2021). Therefore, being able to avoid a long journey to work is perceived as a special benefit of working from home even when one is not sick.

*Proposition 2*: The decision to choose virtual sickness presenteeism is heavily impacted by perceptions of adjustment latitude, especially regarding the latitude to adjust the environment of an individual to meet health-related needs, to adjust temporal work patterns, and to avoid commuting.

### RQ2: How Does the COVID-19 Pandemic Impact the Perception and Formation of Sickness Presenteeism?

We contribute to our understanding of how sickness presenteeism is perceived during a worldwide pandemic and whether this perception will impact sickness presenteeism in the future. As with the reported reasons for virtual sickness presenteeism, we found that previously identified reasons for sickness presenteeism can be transferred to sickness presenteeism during a health crisis. However, two major themes emerged due to the COVID-19 pandemic, namely, the *importance of health* and the *role of rules and regulations*.

While we would not go as far as to state that the COVID-19 pandemic had positive effects, increased awareness regarding health in the workplace can have positive effects. This includes not only an awareness of sickness presenteeism, where health competencies might help to avoid a downward spiral of health but also for health promotion in general, as the health locus of control is an important predictor for taking part in health promotion programs (Rongen et al., 2014). For most organizations, health and health competencies have been in focus during the pandemic. Being able to differentiate between a minor health event and a dangerous infection has been an important aspect of attendance behavior at work (Rongen et al., 2014). Overall, the COVID-19 outbreak, its accompanying protective behaviors, and the health education programs that have sought to improve disease-specific knowledge have impacted the health awareness of employees (Lüdecke et al., 2020). Our participants also reported a change in their perception of health as their interest in the health-related programs and actions taken by their organizations increased. As such, it can be expected that, in the future, employees will expect ongoing discussions about health in the workplace. Plausible changes, such as strict policies regarding protective behaviors (e.g., no longer shaking hands), will not be easily revoked when the pandemic is over, especially if employees perceived them as valuable. As such, expectations regarding the role of the workplace as a healthful environment might carry over after the pandemic.

*Proposition 3*: The COVID-19 outbreak has impacted the perceptions of sickness presenteeism and health in organizations that are likely to carry over into the future because of the increased health awareness of employees.

While we found that most individuals reported no change in rules and regulations, none of those who reported choosing sickness presenteeism stated that those rules and regulations affected their decisions. Furthermore, official rules and regulations were not mentioned in the course of answering questions about a fictitious decision process. This result fits with previous results that have shown that official rules and regulations about absence and presence are, in particular, seldomly successful in influencing absences because of illness, but are more closely related to the absence of work-related motivation (Dalton and Mesch, 1991). Although such a lack of motivation was not the focus of this study, the pandemic might have impacted the perceptions of the rules and regulations related to the correct behavior of sick employees in the future, as some organizations have heavily communicated the importance of following organizational guidelines concerning health. Therefore, employees may now understand better than before that such rules and regulations benefit all members of the organization even when an individual believes that his or her health impairment is manageable, regardless of how this previously led to presenteeism (Miraglia and Johns, 2016). Employees reported that a clear understanding of the rules and regulations helped them to conform. However, future research on the effectiveness of absence policies after the pandemic should determine whether such changes are sustained.

*Proposition 4*: While rules and regulations related to sickness presenteeism tend to be ineffective, the ongoing communication regarding the worth of health-related rules and regulations could serve to reduce sickness presenteeism.

### RQ3: How Does the Connection Between Telecommuting and Virtual Sickness Presenteeism During a Pandemic Impact Individuals and Organizations?

Finally, we found evidence for interactions among the pandemic, telecommuting, and sickness presenteeism. Being “forced” to refrain from on-site sickness presenteeism resulted in virtual sickness presenteeism, which often changed the perception of what it means to be at home. Before the pandemic, being sick at home was perceived as a time when one was unable to work. On the other hand, participants reported that, when they were telecommuting, co-workers expected them to work even when they were ill. Consequently, both telecommuting and the pandemic impacted how sickness presenteeism is perceived in organizations. Although we know of no explicit research on the norms of virtual presenteeism, studies on presenteeism have found that it can lead to extra-time valuation (i.e., the perception that the career of an individual depends on daily working hours), a distrust of supervisors, and competitiveness with co-workers (Ferreira et al., 2015). All of these can be transferred to a virtual work setting. Specifically, aspects such as hidden (virtual) sickness absenteeism, which means not calling in sick and pretending to work virtually, or unobserved sickness presenteeism could create perceptions of distrust, thereby shifting the perceived legitimacy of sickness absenteeism and presenteeism (Ruhle and Süß, 2020) because of the pandemic. For example, the current behavior of being more careful has resulted in an abrupt decline in respiratory disease rates in Germany (Buchholz et al., 2020), which might impact future employees when they consider whether they should work on-site while ill. Overall, both the pandemic and widely expanded telecommuting have impacted the perceptions of norms and, consequently, are likely to change future behavior.

*Proposition 5*: Attendance norms created during the pandemic have impacted virtual sickness presenteeism, (hidden) sickness absenteeism, and on-site presenteeism.

While the possibility of adjusting health-related needs, temporal work patterns, and the volume of work may seem to be only positive changes for the individual, such adjustments could also be detrimental to health based on the context. In addition to results from previous research that highlighted a complex relationship concerning virtual sickness presenteeism, we found evidence for specific phenomena in our sample. For example, employees need *adequate working conditions* when telecommuting, which is not always a given. Non-ergonomic workplaces (e.g., working in bed) increase the risk of several disorders (Buomprisco et al., 2021) and are often prevalent when employees work from home (Davis et al., 2020). Particularly when working from home while ill, our participants reported very specific behaviors to adjust their work to their health, i.e., working in bed or other unusual places to deal with the consequences of the illness. Therefore, even when adequate working conditions are available, virtual sickness presenteeism might be a self-endangering behavior in otherwise favorable working conditions. In addition, especially when virtual sickness presenteeism is undertaken in pursuit of flexibility, employees often use it to push to meet their work goals even if these goals are all but unreachable without the employees being in good health. The outcomes might be negative in terms of creating stress, reducing opportunities to rest adequately, and ultimately prolonging an illness (Dettmers et al., 2016). The notion that employees often opt for sickness presenteeism because they think they are sufficiently robust to deal with the consequences (Lohaus et al., 2020) might be stimulated by the opportunity to work from home.

However, such effects may also depend on the *nature of the health event*. More specifically, the symptoms of some health events that would have led to sickness absenteeism might be related only to issues associated with mobility or being in public but are otherwise unproblematic. In such situations, presenteeism can have positive consequences (Karanika-Murray and Biron, 2020). Therefore, choosing virtual sickness presenteeism might be especially advantageous as it can prevent work from piling. For other kinds of illness, virtual presenteeism might even be beneficial by distracting employees from their illness. Therefore, we propose:

*Proposition 6*: Virtual presenteeism impacts the future health of individuals, but whether this effect is positive or negative depends on the working condition of an individual, the level of adjustment, and the nature of the health event.

## Limitations

Our study has some limitations that should be taken into account when interpreting the findings. First, our results should be considered in light of their origin in Germany. While there is evidence that the decision to choose sickness presenteeism is rooted in the individual, cross-cultural research on sickness presenteeism has revealed large differences in attendance behavior across countries (Ferreira et al., 2019; Reuter et al., 2019). However, aside from general cultural differences that might impact what is perceived as a legitimate reason for absence and presence and the differences in the rules and regulations on the national level, how countries dealt with COVID-19 differed (Hopman et al., 2020; Papageorgiou and Melo, 2020), especially in terms of voluntary and forced telecommuting and the shut-down of workplaces. These differences should be considered (Ruhle et al., 2020), but we are optimistic that our propositions will hold in other cultures. Second, we did not ask additional questions to clarify responses to our open-ended questions or discuss the results and their interpretation with the participants, which is a major strength of qualitative research (Saldaña, 2015). Despite this, it was a limitation rooted in our data-generation process. Therefore, results might be biased by the subjective interpretation of the two authors, although we described our procedures, used adequate measures of reliability when possible, and included various quotations to make our results as transparent as possible. Third, in the group of non-presenteeists, we were unable to separate between individuals that did not have any health events and those that did not choose presenteeism, which might have generated further interesting results. Finally, to avoid overburdening the participants, we asked presenteeists and non-presenteeists different questions. While this approach allowed us to create a broader database, as presenteeists were asked about their behavior and non-presenteeists were asked about a fictitious decision process, we could not compare the results between these two groups. Their answers were closely connected, but we cannot exclude the possibility that the decision processes differed between these two groups.

## Conclusion

The main purpose of this qualitative study was to explore (virtual) sickness presenteeism in the context of the COVID-19 pandemic. We found evidence related to the decision process in choosing virtual sickness presenteeism during a global pandemic and explored the current perceptions of telecommuting and sickness presenteeism. The results of this study indicate that the COVID-19 pandemic and telecommuting have impacted the decision to show absence or presence. The study expands our understanding of virtual sickness presenteeism as a neglected issue in research on attendance behaviors in organizations. We derived propositions that future research could use in examining the consequences of the increase in telecommuting and other consequences of the COVID-19 pandemic. We showed that virtual sickness presenteeism is considered a viable alternative to on-site sickness presenteeism and suggest that future research may analyze the positive and negative consequences of virtual sickness presenteeism.

## Data Availability Statement

The datasets presented in this article are not readily available because the qualitative data includes identifying information that will not be shared with interested researchers. Requests to access the datasets should be directed to Sascha Alexander Ruhle, Sascha.Ruhle@hhu.de.

## Ethics Statement

Ethical review and approval was not required for the study on human participants in accordance with the local legislation and institutional requirements. The patients/participants provided their written informed consent to participate in this study.

## Author Contributions

SR and RS contributed to the conception, design of the study, and analyzed the data. SR organized the database and performed the computation of the reliability. All authors contributed to the manuscript, its revision, and approved the submitted version.

## Funding

We received funding for the open access publication fees from the Open Access Publication Fund of the Heinrich-Heine-University of Düsseldorf.

## Conflict of Interest

The authors declare that the research was conducted in the absence of any commercial or financial relationships that could be construed as a potential conflict of interest.

## Publisher's Note

All claims expressed in this article are solely those of the authors and do not necessarily represent those of their affiliated organizations, or those of the publisher, the editors and the reviewers. Any product that may be evaluated in this article, or claim that may be made by its manufacturer, is not guaranteed or endorsed by the publisher.
